# Understanding the factors associated with married women’s attitudes towards wife-beating in sub-Saharan Africa

**DOI:** 10.1186/s12905-022-01809-8

**Published:** 2022-06-18

**Authors:** Betregiorgis Zegeye, Comfort Z. Olorunsaiye, Bright Opoku Ahinkorah, Edward Kwabena Ameyaw, Eugene Budu, Abdul-Aziz Seidu, Sanni Yaya

**Affiliations:** 1 HaSET Maternal and Child Health Research Program, Shewarobit Field Office, Shewarobit, Ethiopia; 2 Department of Public Health, Arcadia University, Glenside, PA USA; 3 School of Public Health, Faculty of Health, University of Technology Sydney, Sydney, NSW Australia; 4 Department of Population and Health, University of Cape Coast, Cape Coast, Ghana; 5 College of Public Health, Medical and Veterinary Sciences, James Cook University, Townsville, QLD Australia; 6 School of International Development and Global Studies, University of Ottawa, Ottawa, Canada; 7 The George Institute for Global Health, Imperial College London, London, UK

**Keywords:** Wife-beating, Domestic violence, Women’s empowerment, Sub-Saharan Africa, Global health

## Abstract

**Background:**

Intimate partner violence remains a major public health problem, especially in countries in sub-Saharan Africa. We examined the factors associated with married women’s attitudes towards wife-beating in sub-Saharan Africa.

**Methods:**

We used Demographic and Health Survey data of 28 sub-Saharan African countries that had surveys conducted between 2010 and 2019. A sample of 253,782 married women was considered for the analysis. Bivariate and multivariate logistic regression analyses were carried out, and the results were presented using crude odds ratio (cOR) and adjusted odds ratio (aOR) at 95% confidence interval.

**Results:**

The pooled result showed about 71.4% of married women in the 28 countries in this study did not justify wife-beating. However, the prevalence of non-justification of wife-beating varied from 83.4% in Malawi to 17.7% in Mali. Women’s age (40–44 years-aOR = 1.61, 95% CI 1.16–2.24), women’s educational level (secondary school-aOR = 1.47, 95% CI 1.13–1.91), husband’s educational level (higher-aOR = 0.55, 95% CI 0.31–0.95), women’s occupation type (professional, technical or managerial-aOR = 1.66, 95% CI 1.06–2.62), wealth index (richest-aOR = 5.52, 95% CI 3.46–8.80) and women’s decision-making power (yes-aOR = 1.39, 95% CI 1.19–1.62) were significantly associated with attitude towards wife-beating.

**Conclusion:**

Overall, less than three-fourth of married women in the 28 sub-Saharan African countries disagreed with wife-beating but marked differences were observed across socio-economic, decision making and women empowerment factors. Enhancing women’s socioeconomic status, decision making power, and creating employment opportunities for women should be considered to increase women’s intolerance of wife-beating  practices, especially among countries with low prevalence rates such as Mali.

## Background

Violence against women remains a major public health problem [1–4]. The United Nations defines violence against women as “any act of gender-based violence that results in, or is likely to result in, physical, sexual, or mental harm or suffering to women, including threats of such acts, coercion or arbitrary deprivations of liberty, whether occurring in public or private life” [1, 2].

Intimate partner violence (IPV) is one of the most common forms of domestic violence and is manifested in physical, emotional, and sexual forms as defined by the World Health Organization (WHO) [3]. IPV has negative physical (e.g., back pain, limited mobility, injury), sexual (e.g., victims of sexual violence, risky sexual behavior, sexually transmitted infections, including HIV), reproductive (miscarriage, stillbirth, preterm delivery, infant mortality), and mental health (depression, post-traumatic stress, and other anxiety disorders such as sleep difficulties and suicidal attempts) consequences for women and children [2, 4–6]. It is also associated with socioeconomic problems such as isolation, inability to work, loss of wages and lack of participation in regular activities [2, 4–6].

IPV, including wife-beating, is prevalent in all societies across the globe [7, 8]. However, the magnitude and degree to which it is accepted by societies vary across regions; sub-Saharan African countries have higher prevalence of IPV [7, 8]. Out of 15 countries with the highest prevalence of justifying wife-beating in the world, 14 of them are in sub-Saharan Africa (SSA) [8]. About 24% of women in Africa reported that wife-beating is sometimes or always justified [9]. A recent meta-analysis and systematic review study shows that 25.9% of women experienced physical violence or beating by their husbands [10]. Another report in 34 African countries shows that justifying wife-beating varied based on educational level, which is from 41% among individuals with no formal education, to 23% and 25% among individuals who attended primary school and secondary or higher levels of education, respectively [9]. Additionally, justifying wife-beating varied between 25 and 29% among individuals aged 66+ years and 18–25 years, respectively [9].

The attitude of accepting wife-beating as a cultural norm within communities is linked to increased probability of continuation of a low response to wife-beating in societies [11–15]. People who consider wife-beating as a normal phenomenon are less likely to respond timely and support the victims, and the delayed response is less empathetic [16, 17]. Women who justify any form of violence by a husband, including wife-beating usually blame themselves for the violence, are less likely to report the problem to legal authorities, and are more likely to experience long-term psychological problems [15, 18].

Women’s attitude towards wife-beating is a proxy for insight or perception of their status [19, 20] and one indicator of women’s empowerment [21–23]. Evidence shows that women who believe wife-beating is a non-justifiable practice usually are more likely to be aware of their rights, have better self-image and status, and greater sense of empowerment [20, 24, 25]. On the other hand, women who believe wife-beating as justifiable usually believe that a husband has the responsibility of correcting his wife’s behavior even through violent behavior, and those women have less awareness of their rights and less self-image [19, 20, 24, 25]. Hence, refusing or not justifying physical violence against women, including beating by their husbands, is an indicator of empowerment [26, 27].

In May 2016, at the WHO Assembly, several sub-Saharan African countries endorsed a global plan of action to strengthen the role of the health system within a national multispectral response to address interpersonal violence, including wife-beating, against women and girls, and against children [28]. In line with this, some studies in African countries have shown that socioeconomic and demographic factors are associated with wife-beating attitude among women and men [5, 15, 29–32]. However, most of these studies do not reflect recent data (used relatively old data) from (1999–2001) [32], (2003–2007) [33], (2010–2012) [15], and focused only on prevalence [15], or a single country, and among all women of reproductive age [5, 29–31]. To fill these gaps, we aimed to examine the factors associated with married women’s attitude towards wife-beating attitude using nationally representative data from 28 sub-Saharan African countries.

## Methods

### Data source

We extracted nationally representative Demographic and Health Survey (DHS) data conducted between 2010 and 2019 from 28 countries in SSA. DHS is carried out with the financial and technical support of the United States Aid for International Development (USAID) and Inner City Fund (ICF) International [34]. The survey is conducted across several low- and middle-income countries to gather data for monitoring demographic and health indicators, including wife-beating attitude [35].

In DHS, a two-stage stratified cluster sampling technique is applied. In the first stage, enumeration areas (EAs) are selected using probability proportional to size (PPS), and in the second stage, fixed numbers of households (usually 25–30 households) are sampled from the selected EAs using systematic sampling technique [36]. We included 28 countries in SSA based on the inclusion criteria; country with DHS conducted between 2010 and 2019, availability of outcome variable and key explanatory variables (Table 1). We used the individual recode (IR) files for this study. A total of 253,782 married women were included in the analysis. The DHS datasets are freely available for download at https://dhsprogram.com/data/available-datasets.cfm. This manuscript was prepared based on the guidelines for strengthening of observational studies in epidemiology (STROBE) [37].

**Table 1:** Survey year and sampled population across 28 SSA countries

Country	Year of survey	Sampled population (weighted number)	Sampled population (weighted %)
Angola	2015/16	8033	3.2
Burkina Faso	2010	13,392	5.3
Benin	2017/18	11,170	4.4
Burundi	2016/17	9559	3.8
Congo Democratic Republic	2013/14	12,441	4.9
Cote d’Ivoire	2011/12	6448	2.5
Cameroon	2018/19	7463	2.9
Ethiopia	2016	9824	3.9
Gabon	2012	4749	1.9
Ghana	2014	5455	2.1
Gambia	2013	6880	2.7
Guinea	2018	7812	3.1
Kenya	2014	9004	3.5
Comoros	2012	3291	1.3
Lesotho	2014	3609	1.4
Liberia	2013	5875	2.3
Mali	2018	8332	3.3
Malawi	2016/17	15,952	6.3
Namibia	2013	3362	1.3
Nigeria	2018	28,888	11.4
Rwanda	2014/15	6865	2.7
Sierra-Leone	2019	9837	3.9
Senegal	2010/11	10,804	4.2
Chad	2014/15	13,393	5.3
Togo	2013/14	6353	2.5
Uganda	2016	11,379	4.5
Zambia	2018/19	7597	3
Zimbabwe	2015	6015	2.4
Total		253,782	100

### Study variables

#### Dependent variable

The outcome variable for this study was attitude towards wife-beating. In the DHS, women aged 15–49 are asked five questions to measure their attitude towards wife-beating. The questions focus on whether a husband is justified in hitting or beating his wife for at least one of the following five reasons: burning food, arguing with him, going out without telling him, neglecting the children and refusing to have sexual intercourse with him. According to the DHS guideline, a woman is said to disagree to wife-beating if she disagrees with all of these five reasons. Based on this, an overall binary variable was created with a value 1 and 0, where 1 indicated disagreement with all of these reasons, and 0 indicated agreement with at least one of the reasons for wife-beating [38, 39].

#### Independent variables

By referring to several studies [5, 15, 29–32] on women’s attitude towards wife-beating, we included the following independent variables: age in years [15–19, 20–24, 25–29, 30–34, 35–39, 40–44, 45–49]; women’s educational level (no formal education, primary school, secondary school, higher); and husband’s educational level (no formal education, primary school, secondary school, higher). Other explanatory variables included were women’s occupation (no occupation, professional or technical or managerial, agricultural, manual, others); household wealth quintile (poorest, poorer, middle, richer, richest), reading newspaper (no, yes), listening to radio (no, yes), watching television (no, yes), parity (0, 1–2, 3–4, 5+), religion (Christian, Muslim, others), and place of residence (urban, rural).

Other variables were barriers to accessing healthcare services and decision making capacity. Barriers to healthcare services were coded as “no” if the women had no big problems with any of the following four barriers; money needed for treatment, permission of husband to go to health facility, distance to health facility and not wanting to go alone to health facility and coded as “yes” if the women had a big problem with at least one of four barriers. Regarding decision making capacity, we coded as “no” if the women were not involved (either alone or together with their husbands) in all three of the following decision-making parameters: their own health, large household purchases, and to visit families or relatives, and we coded as “yes” if the women were involved in decision making in all three of the above-mentioned decision-making parameters.

### Statistical analyses

Using Stata version-14 software, analysis was done as follows. First, descriptive analysis such as frequency distribution and percentages of the sampled women’s characteristics, and the prevalence of wife-beating attitude were computed. Pearson’s Chi-Square test (χ^2^) was used to test for proportional difference between explanatory variables and prevalence of wife-beating attitude and, using bivariate logistic regression analysis, we examined the crude odds of each explanatory variable with the prevalence of wife-beating attitude (disagreement with wife-beating). Then, multicollinearity test was conducted to assess the presence of collinearity among the explanatory variables using the Variance Inflation Factor (VIF); we found no significant evidence of collinearity (VIF Mean = 1.87, VIF Min = 1.09, Max VIF = 3.43). Finally, all statistically significant explanatory variables in the bivariate regression test were entered into a multivariable logistic regression model. DHS data has weight variables in each country’s data, and we took these variables into account in the pooled statistical analysis. We first generated weight in each of the country’s dataset, using the weight variables, before we pooled the data together. Thereafter, we executed the “svyset” command in the pooled dataset including the unique codes for each country's population sample unit (PSU) and strata. The adequacy of the final model was checked by Hosmer–Lemeshow Test, and it showed that the model was a good fit (*p* value = 0.5947). The results were presented using crude odds ratio (cOR) and adjusted odds ratio (aOR), at a 95% Confidence Interval (CI). Any P-value less than or equal to 0.05 (*p* ≤ 0.05) was considered statistically significant.

### Ethical clearance

The data used for the analysis of this study was secondary data that are publicly available (https://dhsprogram.com/data/available-datasets.cfm). Since ethical procedures were the responsibility of the institutions that funded, commissioned, and managed the surveys, further ethical clearance was not required. ICF international ensures that all the DHS surveys follow the U.S. Department of Health and Human Services rules for respecting of human subjects’ rights. For more details related to ethical issues, readers can visit http://goo.gl/ny8T6X.

## Results

### Socio-demographic characteristics of respondents

As shown in Table 2, a total of 253,782 married women were included in the analysis for this study. Among the study participants, about 7.9% were adolescents (15–19 years) and 35.3% lived in rural areas. About 27.5% and 21.1% of the participants and their husbands, respectively, had no formal education, and 25.3% of participants were not working. Approximately 69.5% of participants encountered barriers to accessing healthcare services, and 34.6% of participants did not decide, either alone or together with their husband, on any of the three decision making parameters—their own health, purchasing large household expenses and visiting families/relatives.

**Table 2:** Socio-demographic characteristics and prevalence of disagreement with wife-beating across explanatory variables, 28 SSA countries

Variables	Frequency (weighted %)	Disagreed with wife-beating (71.4%)		Chi-square, *p* value
		No, weighted %	Yes, weighted %	
*Age in years*				χ^2^ = 66.06, *p* < 0.001
15–19	15,381 (7.86)	41.63	58.37	
20–24	40,182 (19.88)	30.02	69.98	
25–29	49,269 (21.60)	26.68	73.32	
30–34	42,258 (16.88)	25.75	74.25	
35–39	35,798 (14.56)	28.56	71.44	
40–44	25,700 (11.73)	26.11	73.89	
45–49	19,483 (7.51)	27.15	72.85	
*Women’s educational level*				χ^2^ = 298.25, *p* < 0.001
No formal education	99,491 (27.46)	37.69	62.31	
Primary school	75,431 (38.90)	32.12	67.88	
Secondary school	45,666 (29.65)	18.37	81.63	
Higher education	7473 (3.98)	7.77	92.23	
*Husband’s educational level*				*χ*^2^ = 176.65, *p* < 0.001
No formal education	86,384 (21.13)	31.8	68.2	
Primary school	57,989 (26.62)	37.01	62.99	
Secondary school	57,884 (44.91)	24.58	75.42	
Higher education	14,720 (7.34)	13.52	86.48	
*Currently working*				χ^2^ = 8.07, *p* = 0.0647
No	87,517 (25.30)	26.14	73.86	
Yes	165,956 (74.70)	29.44	70.56	
*Wealth index*				χ^2^ = 588.73, *p* < 0.001
Poorest	51,782 (17.92)	41.51	58.49	
Poorer	47,375 (20.66)	39.99	60.01	
Middle	45,144 (20.72)	33.71	66.29	
Richer	42,578 (20.59)	18.68	81.32	
Richest	41,192 (20.11)	10.3	89.7	
*Media exposure*				χ^2^ = 119.22, *p* < 0.001
No	108,957 (28.22)	37.38	62.62	
Yes	144,352 (71.78)	25.15	74.85	
*Parity*				χ^2^ = 12.84, 0.0701
0	14,920 (4.00)	28.58	71.42	
1–2	69,399 (29.49)	26.59	73.41	
3–4	63,805 (28.82)	27.75	72.25	
5+	79,947 (37.69)	30.83	69.17	
*Decision making*				χ^2^ = 84.19, *p* < 0.001
No	127,476 (34.59)	34.97	65.03	
Yes	90,356 (65.41)	25.24	74.76	
*Religion*				χ^2^ = 16.37, *p* < 0.01
Christian	143,579 (93.53)	28.07	71.93	
Others	109,958 (6.47)	36.36	63.64	
*Barriers to accessing healthcare*				χ^2^ = 33.4496, *p* < 0.01
No	70,270 (30.5)	24.2	75.8	
Yes	138,869 (69.5)	30.54	69.46	
*Place of residence*				χ^2^ = 248.49, *p* < 0.001
Urban	72,778 (64.71)	22.73	77.27	
Rural	155,293 (35.29)	39.37	60.63	

### Prevalence of wife-beating attitude

**Fig. 1:**
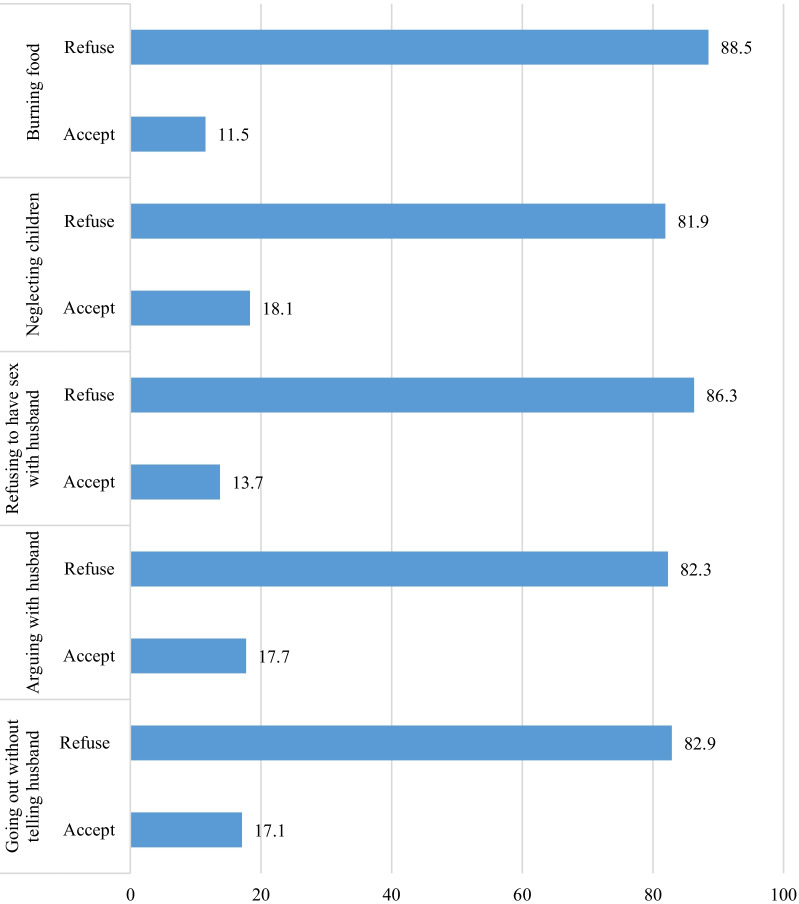
Percentage distribution of wife beating attitude among married women by reasons for wife beating in 28 sub-Saharan African countries: evidence from Demographic and Health Survey

The pooled result shows that about 71.4% of married women in the 28 countries in SSA disagreed with wife-beating for all the five reasons; going out without telling husband, arguing with husband, neglecting the children; refusing to have sex with husband, and burning food. As shown in Fig. 1, large proportions of married women (88.5%) disagreed with wife-beating when the wife burns food, when the wife refuse to have sex with husband (86.3%), goes out without telling husband (83%), arguing with husband (82.3%) and neglecting children (82%) (Fig. 1).

### Prevalence of wife-beating across studied countries

**Fig. 2:**
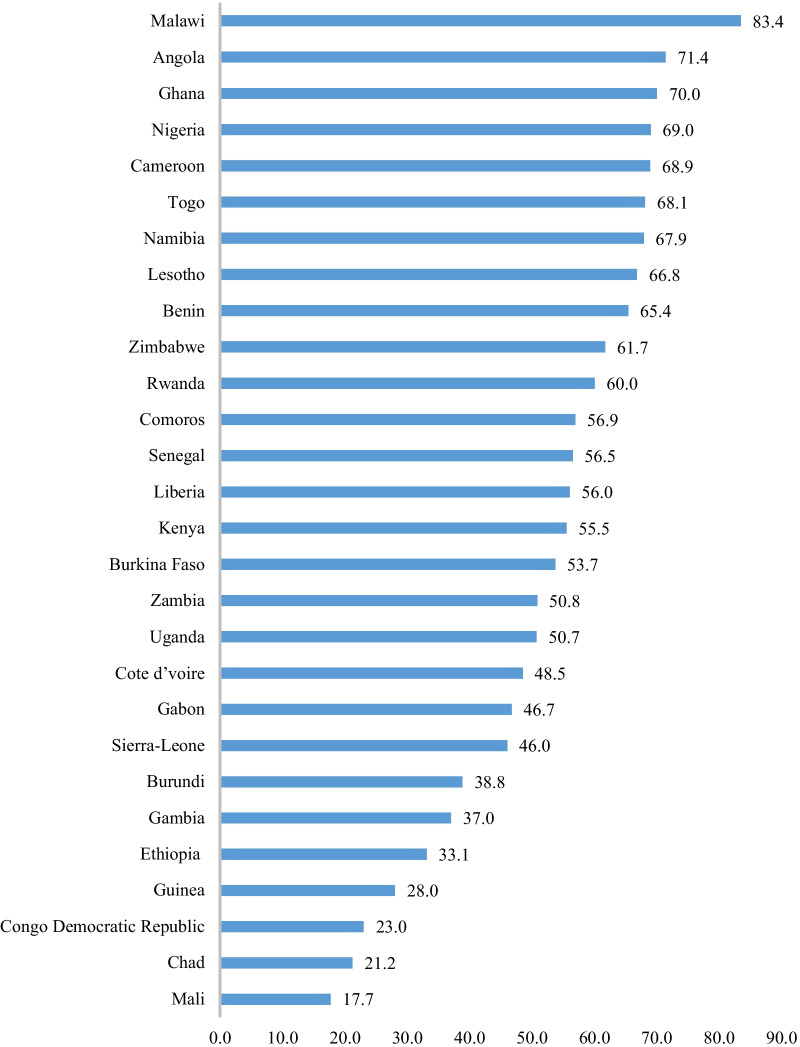
Prevalence of disagreement with wife beating among married women in 28 sub-Saharan African countries. Evidence from Demographic and Health Survey

As shown in Fig. 2, the highest prevalence of disagreement with wife-beating was seen in Malawi (83.4%), Angola (71.4), Ghana (70%), Cameroon (68.9%), Togo (68.1%) and Benin (65.4%) respectively. On the other hand, the lowest prevalence of disagreement with beating was reported in Mali (17.7%), Chad (21.2%), Congo Democratic Republic (23%), Guinea (28%) and Ethiopia (33.1%) respectively (Fig. 2).

### Prevalence of wife-beating attitude across explanatory variables

Table 2 shows variations in the prevalence of wife-beating attitude (disagreed/not justified for wife-beating) across the explanatory variables’ subgroups. For instance, about 58.4% of married women who were within the age groups 15–19 years disagreed with wife-beating; while 74.3% of married women who were within the age groups of 30–34 years disagreed with wife-beating. The prevalence varied from 62.3 to 92.2% between not educated and those married women who attended higher education respectively. Similarly, there were 61.5–90.8% variations in prevalence of wife-beating attitude (disagreed with wife-beating) between married women with professional/technical/managerial and agricultural occupation types respectively (Table 2).

### Factors associated with wife-beating attitude

As shown in Table 3, higher odds of disagreement with wife-beating was seen among married women who were within the age groups of 45–49 years (aOR = 1.50, 95% CI 1.07–2.11), 40–44 (aOR = 1.61, 95% CI 1.16–2.23),  30–34 years (aOR = 1.48, 95% CI 1.12–1.96), 25–29 years (aOR = 1.41, 95% CI 1.09–1.82) and 20–24 years (aOR = 1.34, 95% CI 1.04–1.73) as compared to (15–19 years) married adolescents. Similarly, the results showed higher odds of disagreement with wife-beating among married women who attended secondary school (aOR = 1.54, 95% CI 1.20–1.97) and higher (aOR = 2.50, 95% CI 1.17–5.33) as compared to those who had no formal education. Moreover, higher odds of disagreement with wife-beating was seen among married women who were from richest (aOR = 5.65, 95% CI 3.65–8.77), richer (aOR = 3.18, 95% CI 2.17–4.66) and middle (aOR = 1.51, 95% CI 1.12–2.04) households as compared to married women who were from poorest households. In addition, higher odds of disagreement with wife-beating was seen among married women who had decision making capacity (aOR = 1.38, 95% CI 1.19–1.61) as compared to those married women who had no decision making capacity. On the contrary, we found lower odds of disagreement with wife-beating among married women whose husbands attended primary (aOR = 0.78, 95% CI 0.65–0.94) and secondary school (aOR = 0.75, 95% CI 0.62–0.92) as compared to married women whose husbands did not have formal education.

**Table 3:** Bivariate and multivariate logistic regression results for factors associated with disagreement with wife-beating among married women: evidence from DHSs of 28 SSA countries

Variables	cOR (95% CI)	aOR (95% CI)
*Age in years*		
15–19	Ref	Ref
20–24	1.66 (1.30–2.11)***	1.34 (1.04–1.73)*
25–29	1.95 (1.52–2.51)***	1.41 (1.09–1.82)**
30–34	2.05 (1.55–2.71)***	1.48 (1.12–1.96)**
35–39	1.78 (1.37–2.31)***	1.33 (0.99–1.79)
40–44	2.01 (1.46–2.78)***	1.61 (1.16–2.23)**
45–49	1.91 (1.38–2.64)***	1.50 (1.07–2.11)*
*Women’s educational level*		
No formal education	Ref	Ref
Primary school	1.27 (1.06–1.53)**	1.11 (0.92–1.35)
Secondary school	2.68 (2.21–3.26)***	1.54 (1.20–1.97)**
Higher education	7.17 (3.70–13.90)***	2.50 (1.17–5.33)*
*Husband educational level*		
No formal education	Ref	Ref
Primary school	0.79 (0.65–0.95)*	0.78 (0.65–0.94)*
Secondary school	1.43 (1.19–1.71)***	0.75 (0.62–0.92)**
Higher education	2.98 (1.87–4.74)***	0.58 (0.33–1.00)
*Currently working*		
No	Ref	
Yes	0.84 (0.71–1.01)	–
*Wealth index*		
Poorest	Ref	Ref
Poorer	1.06 (0.87–1.30)	1.12 (0.91–1.39)
Middle	1.39 (1.08–1.80)*	1.51 (1.12–2.04)**
Richer	3.08 (2.28–4.17)***	3.18 (2.17–4.66)***
Richest	6.17 (4.44–8.58)***	5.65 (3.65–8.77)***
*Media exposure*		
No	Ref	Ref
Yes	1.77 (1.51–2.08)***	0.85 (0.73–1.01)
*Parity*		
0		Ref
1–2	1.10 (0.78–1.56)	–
3–4	1.04 (0.73–1.47)	–
5 +	0.89 (0.64–1.25)	–
*Decision making*		
No	Ref	Ref
Yes	1.59 (1.37–1.84)***	1.38 (1.19–1.61)***
*Religion*		
Christian	Ref	Ref
Others	0.68 (0.52–0.89)	0.91 (0.69–1.19)
*Barriers to accessing healthcare service*		
No	Ref	Ref
Yes	0.72 (0.60–0.87)**	0.93 (0.77–1.12)
*Place of residence*		
Urban	Ref	Ref
Rural	0.45 (0.36–0.55)***	1.07 (0.81–1.43)

*Ref* reference categories, *significant at *p* < 0.05, **significant at *p* < 0.01, ***significant at *p* < 0.001, *aOR* adjusted odd ratio, *cOR* crude odd ratio

## Discussion

In this study, we examined the prevalence of disagreement with wife-beating and its associated factors among married women in 28 sub-Saharan African countries. The findings show that about 71.4% of married women in the  studied countries disagreed with all the five wife-beating reasons. The prevalence varied across countries, from 83.4% in Malawi to 17.7% in Mali. Our findings are supported by previous literature in SSA [10]. The variation in the prevalence of disagreement with wife-beating across countries could be linked to disparities in women’s educational achievements and cultural norms [15, 40]. Moreover, contextual factors [41], including socioeconomic disparities across countries [10, 42–44] may explain variations in the prevalence of disagreement with wife-beating.

The study shows that woman’s age, women’s educational level, husband’s educational level, women’s occupation type, economic status and decision-making power were significantly associated with disagreement with wife-beating among married women. More specifically, we found higher odds of disagreement with wife-beating among older married women as compared to younger women. These findings are comparable with previous studies in 39 LMIC [15], Jordan [11], Nigeria [30], and Bangladesh [44]. The acceptance of wife-beating may be linked with cultural beliefs [5, 45, 46]. Higher odds of disagreement with wife-beating among older women might be due to the long duration of living within the communities long relationship between women and their husbands, which  allow women to understand their important role within the union and have better awareness about societal culture and norms [30].

In this study, higher odds of disagreement/not justifying wife-beating were seen among married women who attended secondary school compared to married women who had no formal education. Consistent findings were documented in Ethiopia [29, 31], Nigeria [30], SSA [32] and 39 LMIC [15]. This could be partly explained by the associations of women’s education with employment opportunities and income-gaining capacities [8]. Women’s education shapes community discourse and affects women’s perception within the society [8]. In the African context, women’s education is also linked with political knowledge, participation, and decision-making capacities [8, 47]. Education is known to positively affect women’s behavior and disagreeing attitude toward wife-beating because they learn about and have greater differentiation capacities between the actual societal norms and the global context regarding women violence [8, 48], and education increases their exposure to global discourse that again raise their capacity in rejecting partner violence [8, 48]. Previous study in Kenya on scholarship programs targeted to increase girls’ secondary schooling [49] and another study in Sierra Leone focused on policy reform to expand primary school coverage confirmed that girls’ or women’s education reduces acceptance for wife-beating [50]. However, some scholars argue that women with higher educational attainment may face higher risk of beating by their husbands because educated women are more likely to challenge norms and cultures that support male dominance [8, 41].

In contradiction with a previous study in Ethiopia [31], we found lower odds of disagreement with wife-beating among married women with educated husband as compared to married women whose husbands had no formal education. Further qualitative studies would be beneficial in investigating the reasons for lower odds among married women with educated husbands as compared to non-educated husbands.

Moreover, we found that higher odds of disagreeing/not justifying wife-beating among married women with professional, technical or managerial occupation types as compared to married women who were not working. Comparable findings were reported in SSA [8] and in a study conducted by WHO [51]. Beyond its positive effect on income and wealth, employment is also protective against abuse [8, 51].

The study also shows higher odds of disagreement with wife-beating among married women in higher economic status as compared to married women in lower economic status. This finding is consistent with previous studies conducted in Ghana [5], Ethiopia [29], Nigeria [30] and LMICs [15]. A plausible explanation could be women in wealthier households are more likely to be educated and have exposure to media that allow them to prefer discussion and democratic ways to any conflicting issues instead of reacting and arguing with their husbands [30]. Again, they might not have shortage of finance and related conflict causing situation [30]. Unlike wealthier women, poorer women may interpret wife-beating as normal life or accept it due to their economic dependence on their husbands [52, 53]. It is not surprising that justifying or tolerating physical violence (including beating) is linked with independence, as  poor women who may be independent may justify wife beating due to their dependence on their husband [54]. Studies in SSA also confirmed associations between economic status and wife-beating [52, 55, 56].

Decision-making power of married women was associated with wife-beating attitude [29]. We found higher odds of disagreeing/not justifying wife-beating among married women with decision making power compared to married women who were not involved in decision-making. Previous studies in Ethiopia [29] and Nigeria [30] reported similar findings. It is possible that women who had decision-making power would have opportunities to move and access information through their social networks [57–59]. Decision making power is an important concept for the woman herself and the society because the combination of access capabilities and actions shape whether women have influence over the decisions about their private lives, their health, and the health of their children and family [60].

### Strengths and limitations of the study

Investigating wide-ranging socioeconomic, demographic, and women’s empowerment factors using a large sample of nationally representative data across several countries in SSA is a major strength of the paper. However, the findings should be interpreted in the context of the following limitations. First, we used data from DHSs that were conducted within nine years; comparison of findings might not be always possible due to the time effect. Second, though we included most countries, still there are few countries in SSA that were excluded from the analysis because of the exclusion criteria; as a result, generalizing the findings to all countries in SSA may not be possible. Third, due to the cross-sectional nature of DHS data, establishing a cause-effect relationship may not be possible. Finally, the survey participant’s self-reported data may be affected by recall bias.

## Conclusions and policy implications

Overall, less than three-fourths of married women in the 28 sub-Saharan African countries disagreed with wife-beating but marked differences were observed across socio-economic, decision making and women empowerment factors. Women’s age, women’s educational level, husband’s educational level, women’s occupation, wealth index and women’s decision-making power were significantly associated with wife-beating attitude.

Changing societies’ (including women’s) attitude toward wife-beating required political commitment and integrated implementations of policies and programs among different sectors such as Ministries of Health and Justice, and Ministries of Child and Women or Gender and Social Affairs. Policy makers need to design policies and strategies that enhance women’s socioeconomic status including creating employment opportunities and their decision-making capacity to increase women’s intolerance of wife-beating practices, especially among countries with low prevalence rates such as Mali.

Though further studies on husband education and married women’s attitude towards wife-beating may be needed, these findings highlight that awareness creation for general population and involvement of educational courses about wife-beating practice’s negative consequences to reduce challenges from their husband and the general community.

Moreover, policy and regulation transformation into actions through the development of service delivery protocols or guidelines are required for better achievement in the reduction of wife-beating practices and societal acceptance attitude.

## Data Availability

Data for this study were sourced from Demographic and Health surveys (DHS) and available here: http://dhsprogram.com/data/available-datasets.cfm.

## Abbreviations

AOR: Adjusted odd ratio
COR: Crude odds ratio
DHS: Demographic and health surveys
IPV: Intimate partner violence
SSA: Sub-Saharan Africa
VIF: Variance inflation factor
